# Multidisciplinary Simulation of Trauma in Pregnancy with Resuscitative Endovascular Balloon Occlusion of the Aorta (REBOA) Utilization

**DOI:** 10.7759/cureus.32820

**Published:** 2022-12-22

**Authors:** Peter Hopmann, Jaya Sai Varre, Gary Duncan, William B Devoe, Brad D Gable

**Affiliations:** 1 General Surgery, OhioHealth Riverside Methodist Hospital, Columbus, USA; 2 Medical Education and Simulation, OhioHealth Riverside Methodist Hospital, Columbus, USA; 3 Medical Simulation, OhioHealth Riverside Methodist Hospital, Columbus, USA

**Keywords:** cesarean section, reboa, medical education, trauma, simulation

## Abstract

Background

Studies have demonstrated the use of resuscitative endovascular balloon occlusion catheters of the aorta (REBOA) in the setting of postpartum hemorrhage and traumatic hemorrhagic shock. However, REBOA is infrequently utilized leading to a lack of clinician comfort. This study’s aim was to demonstrate the utility of REBOA in a hemorrhaging pregnant trauma patient and improve clinician comfort with the placement of REBOA while emphasizing collaboration between medical specialties.

Methods

A multidisciplinary in-situ simulation was developed for the management of a pregnant patient with an abdominal gunshot wound evaluated by obstetrics and surgery teams. A trauma survey, emergency c-section, massive transfusion protocol (MTP), and evaluation for and placement of REBOA were indicated during the simulation. A standardized Return on Learning questionnaire was utilized to determine participants' reactions and confidence gained during the simulation.

Results

A total of 32 of 41 participants completed the survey (78%). A statistically significant increase in confidence was reported in the ability to prioritize the care of a pregnant patient with hemorrhagic shock (p = 0.016), apply MTP to the appropriate clinical setting (p = 0.03), and analyze critical decisions made for abdominal trauma in pregnant patients (p = 0.006). Specifically for physicians, a significant increase in confidence in the ability to identify indications/contraindications for REBOA placement in hemorrhaging patients was observed (p = 0.021).

Conclusions

A multidisciplinary simulation for the management of a pregnant patient in hemorrhagic shock secondary to penetrating abdominal trauma improved learner confidence in MTP, care of pregnant patients in hemorrhagic shock, and abdominal trauma in pregnancy. Physician learners gained confidence in indications for REBOA placement in abdominal trauma. This simulation was highly relevant to all participants.

## Introduction

Resuscitative endovascular balloon occlusion of the aorta (REBOA) has been shown to have increasing utility in both obstetrics and trauma surgery. In trauma, hemorrhage is the most common cause of preventable deaths, necessitating the importance of rapid hemorrhage control in the setting of major vessel injury or solid organ injury in the thoracic and abdominopelvic cavities [1]. Trauma-related injury has also been shown to be the number one cause of non-obstetric maternal death [2]. In obstetrics, hemorrhage is the leading cause of death in pregnant women worldwide, of which the majority is postpartum hemorrhage [2]. Multiple studies including case reports and larger systematic reviews have demonstrated that the use of REBOA is associated with reduced hemorrhage and reduced blood product transfusions, particularly in the setting of invasive placental disorders such as placenta accreta [1,3-7]. Multiple simulation studies have been published in both obstetrics and trauma surgery regarding the usage of REBOA in each respective field, and there have also been simulations regarding trauma in pregnancy [8-13]. Specifically, no reported simulation-based education for the use of REBOA in traumatically injured pregnant patients was identified in the literature review. The ability of REBOA to potentially reduce morbidity and mortality was discussed during the debriefing session to improve learner knowledge. The aim of this simulation study was to demonstrate the utility of REBOA in pregnant trauma patients and improve clinician comfort with the placement of REBOA.

REBOA is a minimally invasive method of obtaining control of massive hemorrhage via common femoral access, followed by the placement of an occluding balloon catheter within the aortic lumen. The area of confirmed or suspected hemorrhage dictates the aortic zone of deployment which is as follows: Zone 1 (supraceliac/descending aorta), Zone 2 (suprarenal/pararenal), and Zone 3 (infrarenal/superior to aortic bifurcation). Depending on the technique, either complete or partial aortic balloon occlusion is achieved in Zone 1 or Zone 3 with the restoration of systolic blood pressure near 90 mmHg. Prior research has demonstrated placement without fluoroscopy to be safe and performed with a high level of technical success [14]. REBOA has been shown to be useful in trauma situations, pregnant patients with invasive placental disorders, and even in pregnant trauma [1-7]. There is also emerging evidence that REBOA may decrease blood loss without compromising fetal outcomes [15].

In-situ simulations have shown to be effective teaching methods for high-risk, low-frequency clinical scenarios so that learners have exposure to these situations and can learn from mistakes without compromising patient care. Pregnancy-related trauma and emergent cesarean sections are two areas where simulation scenarios have proven to be effective [8,16-18]. Introducing multiple disciplines to a simulation can help improve communication and inter-departmental perceptions [19,20]. This has shown benefits not only between residency specialties, but also improves collaborative attitudes between residents and nursing staff [21,22]. In-situ simulations allow participants to experience a simulated case in the physical environment where they may encounter a true patient scenario [23]. As a result of in-situ simulations, learners have a better understanding of the layout of the room(s), where needed supplies may be located, and processes and protocols utilized in the clinical environment [24]. It was hypothesized that a multidisciplinary in-situ simulation would improve learners' confidence in stated objectives related to trauma, pregnancy, and REBOA utilization.

## Materials and methods

At this institution obstetric and gynecology (OBGYN) and general surgery residents perform bi-annual interdisciplinary simulation education. The group of expert educators from OBGYN, surgery, and medical simulation discussed how simulation-based education could meet current educational needs. The expert educators identified an opportunity for education about traumatically injured pregnant patients. Specifically, learners likely were aware of the use of REBOA within their specialties but could benefit from discussion of this intervention when patients are being acutely assessed by both surgical and obstetric services.

Objectives for this simulation were as follows (Table 1):

**Table 1:** Simulation Objectives REBOA: Resuscitative Endovascular Balloon Occlusion of the Aorta

By the End of the Course, Learners Will Be Able To:
Prioritize the care of a pregnant patient with hemorrhagic shock from hemoperitoneum
Demonstrate interdisciplinary collaboration with other specialties
Apply massive transfusion protocol (MTP) to the appropriate clinical settings
Analyze critical decisions made for abdominal trauma in pregnant female patients
Identify indications/contraindications for REBOA placement in hemorrhaging patient

It was determined that to best accomplish these goals an in-situ simulation within the emergency department trauma bay would be most beneficial. To that end, all local protocols would be followed including notification of the appropriate emergency department, trauma, and obstetric teams. The in-situ simulation scenario included participation from one emergency department attending physician, three general surgery residents, two OBGYN residents, as well as emergency room (ER) nurses, respiratory therapy, pharmacy, radiology technologists, blood bank, operating room staff, neonatal intensive care unit staff, labor and delivery nurses, social work, pastoral care, and emergency medical transport staff. Learners were divided into the active simulation group, which participated in the formal trauma scenario, and an observer group, which watched the events of the simulation via video feed. The simulation was then repeated for the second group of learners.

The simulated scenario was a 28-year-old female gravida 2 para 1 (G2P1) at 36 weeks gestation who presented to the emergency department as a level 1 trauma activation after a gunshot wound to the right upper abdomen following a domestic dispute. En route, the patient complained of severe right upper quadrant pain with blood loss noted in the field. She did not fall or hit her head. Initial vital signs showed the patient was normotensive with a blood pressure of 135/80, however, she was noted to be hypotensive to 80/45 and tachycardic to a heart rate of 120s on arrival to the trauma bay. OBGYN and general surgery were called to evaluate the patient in the trauma bay using standard notification protocols.

The surgery team subsequently performed a standard trauma evaluation following current Advanced Trauma Life Support (ATLS 10th edition) guidelines including an appropriate primary survey and adjunctive testing which included a focused assessment with sonography for trauma (FAST) examination. The OBGYN team evaluated the fetus by obtaining fetal heart tones. These examinations revealed the patient to be in extremis with active bleeding including blood noted in the right upper quadrant on the FAST exam, and fetal heart tones with decelerations indicating fetal distress. The progression of the trauma scenario to various branch points was based on how quickly and accurately the teams identified these problems and developed a treatment plan. The ultimate goal was for the OBGYN team to determine the need for emergent cesarean section in the trauma bay, while the surgery team coordinated resuscitation with massive transfusion protocol (MTP), the need for the placement of REBOA for hemorrhage control, followed by definitive management of the patient’s traumatic hemorrhage in the operating room. Development of the case scenario, including initial brainstorming meeting, literature review, case write-up and formatting, and simulation walk-through took approximately 12 man-hours. The full case scenario is provided in Appendix A.

Following completion of the 15-minute trauma scenario, all 41 participants took part in a 45-minute structured discussion led by debriefing experts. All participants were then given a questionnaire in which they were asked to rate their confidence level of performing various tasks related to the simulation before the simulation, and after the simulation and debriefing session was complete. This questionnaire was based on Phillips’ Return on Investment model for training evaluation [25] which is used by the authors’ institution as a standardized quality improvement measure. This questionnaire is used for all quality improvement activities within the hospital system and adapted to meet the specific learning objectives. Levels studied with this simulation education were Level 0 (participants/input), Level 1 (reaction/relevance), and Level 2 (knowledge). Objectives included the ability to prioritize the care of the pregnant patient with hemorrhagic shock, the ability to demonstrate interdisciplinary collaboration with other specialties, the ability to apply MTP, the ability to analyze critical decisions made for abdominal trauma in pregnant patients, and to identify indications/contraindications for REBOA placement in patients with ongoing hemorrhage. The survey was given at the end of the simulation with prompts for learner confidence before and after the course. The survey questionnaire also included components regarding whether the simulation was relevant to current work, provided new information, or clarified existing information, whether the simulation was realistic, and options to provide suggestions for improvement. For data analysis, the learners’ confidence and responses to the evaluation questions were assigned a numeric score (1 - strongly disagree, 2 - disagree, 3 - neutral, 4 - agree, 5 - strongly agree). Statistical analysis was performed using Statistical Product and Service Solutions (SPSS) (IBM SPSS Statistics for Windows, Version 25.0, Armonk, NY) based on traditional two-sided t-tests with alpha error set at 5% and 95% confidence intervals.

Finally, a REBOA insertion simulation station was made available to all providers for practice with a trained representative.

The equipment utilized for the simulation was crafted using a Noelle ® Maternal Care Simulator (Gaumard Scientific, USA) as a base. Within the manikin, bowels were created using a two-inch casting stockinette soaked in a latex coating and submerged in water prior to the simulation to simulate the texture and density of intestines. Uterus was created with a silicone casing housing a child’s plastic doll and filled with water to mimic amniotic fluid when opened surgically. Because the REBOA model available at the hospital could not be housed within the Noelle model, a substitute model was created. This was done with one-half-inch diameter rubber tubing that was clamped within the manikin’s right thigh to simulate a femoral artery that extended into the abdominal cavity to represent the aorta and allow the REBOA catheter to be extended into the manikin during the simulation. A one-way valve was placed at the caudal end of the tubing with a bulb and tubing filled with a blood substitute. During the simulation, a confederate at the head of the bed pulsed the bulb to simulate a femoral pulse which was able to be palpated by the learner during the simulation. Tubing was covered with Dragon Skin (™) silicone at the groin for palpation and insertion of the femoral line. A 3D-printed silicone liver model, which had been used in a previous simulation, was placed in the abdominal cavity as well [21]. This had placement of intravenous (IV) tubing that was also controlled by a confederate at the head of the bed to show bleeding from the liver. Additional blood substitute was poured into the abdomen prior to the simulation to show hemoperitoneum present upon opening the abdomen. The abdominal cavity was covered with simulated abdominal musculature and skin for surgical opening. Two IV fluid-compatible arms were secured to the existing manikin arms to facilitate peripheral IV access and the ability to realistically push fluids and blood products during the simulation. Figures showing the manikin pre- and post-simulation available in the Appendices section. The approximate cost of the model for this simulation was reduced by reusing components from previous simulations, with the utilization of available materials and the use of the previously purchased Noelle ® model. The total cost for new materials was less than $25. Assembly time for the model including the creation of the bowels, uterus, and femoral artery components, planning sessions, and movement of materials between simulation sites was approximately 22 man-hours.

## Results

**Figure 1:**
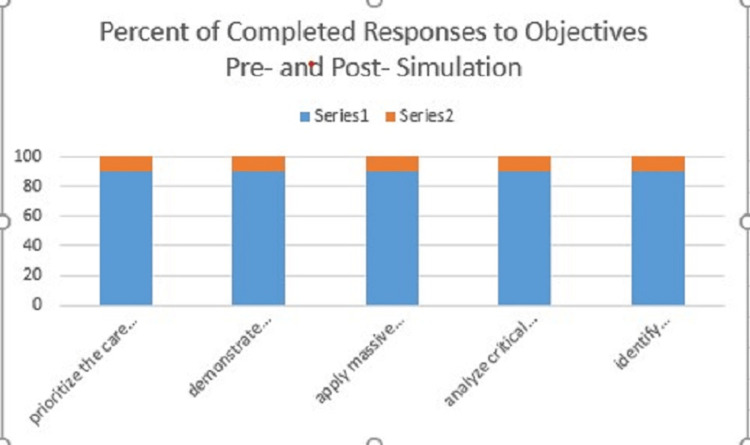
Percent of Completed Objectives for Pre- vs Post-Simulation Questionnaire Series 1 - Post-Simulation Series 2 - Pre-Simulation

A total of 41 participants participated in the simulation (or observed via live-stream video) and debriefing, and 32 fully completed the post-education survey (response rate 78%). Thirty-seven participants completed the pre-simulation portion of the survey but did not complete it in full (Figure 1). Participants included general surgery residents (n=7, 18.9%), OBGYN residents (n=14, 37.8%), attending physicians (n=1, 2.7%), trauma advanced practice providers (n=3, 8.1%), ER nurses (n=6, 16.2%), OB surgical technicians (n=3, 8.1%), and other staff including one medical student, family medicine resident, and a pharmacist. Five surveys were incomplete and so were omitted from data analysis.

A statistically significant increase in overall confidence was reported in the following competencies: ability to prioritize the care of a pregnant patient with hemorrhagic shock from hemoperitoneum (pre-simulation average - 3.76 and post-simulation average - 4.12, p = 0.016, 95% confidence interval (CI) = 0.077-0.65), ability to apply MTP to the appropriate clinical setting (pre-simulation average - 3.70 and post-simulation average - 3.88, p = 0.03, CI = 0.02-0.34), ability to analyze critical decisions made for abdominal trauma in pregnant female patients (pre-simulation average - 3.61 and post-simulation average - 3.94, p = 0.006, CI = 0.11-0.56) (Table 2). When focusing on physicians (residents/attendings) there was a significant increase in confidence regarding the ability to identify indications/contraindications for REBOA placement in hemorrhaging patients (pre-simulation average - 2.85 and post-simulation average - 3.35, p = 0.02, CI = 0.09-0.91) (Table 3).

**Table 2:** Pre-/Post-Survey Responses for All Participants' Confidence in Objectives Score on a 5-point Likert scale (1 - Strongly Disagree to 5 - Strongly Agree) All objectives phrased, "I am confident in my ability to ... " REBOA - Resuscitative Endovascular Balloon Occlusion of the Aorta

Objectives	Pre-Simulation Confidence Mean (Median)	Post-Simulation Confidence Mean (Median)	p-Value	95% Confidence Interval
Prioritize the care of a pregnant patient with hemorrhagic shock from hemoperitoneum	3.76 (4.00)	4.23 (4.00)	0.016	0.077-0.650
Demonstrate interdisciplinary collaboration with other specialties	4.03 (4.00)	4.15 (4.50)	0.21	-0.069-0.31
Apply massive transfusion protocol (MTP) to the appropriate clinical settings	3.70 (4.00)	3.88 (4.00)	0.03	0.020-0.340
Analyze critical decisions made for abdominal trauma in pregnant female patients	3.61 (4.00)	3.94 (4.00)	0.006	0.110-0.560
Identify indications/contraindications for REBOA placement in hemorrhaging patient	2.91 (3.00)	3.24 (3.00)	0.054	-0.001-0.66

**Table 3:** Pre-/Post-Simulation Responses, Physician Only Score on a 5-point Likert scale (1 - Strongly Disagree to 5 - Strongly Agree) All objectives phrased, "I am confident in my ability to ... "

Objectives	Pre-Simulation Confidence Mean (Median)	Post-Simulation Confidence Mean (Median)	p-Value (Mean)	95% Confidence Interval (Mean)
Prioritize the care of a pregnant patient with hemorrhagic shock from hemoperitoneum	4.1 (4.00)	4.2 (4.00)	0.54	-0.23-0.43
Demonstrate interdisciplinary collaboration with other specialties	4.1 (4.00)	4.25 (4.50)	0.27	-0.12-0.42
Apply massive transfusion protocol (MTP) to the appropriate clinical settings	3.7 (4.00)	3.9 (4.00)	0.10	-0.04-0.44
Analyze critical decisions made for abdominal trauma in pregnant female patients	3.75 (4.00)	4.0 (4.00)	0.096	-0.04-0.54
Identify indications/contraindications for REBOA placement in hemorrhaging patient	2.85 (3.00)	3.35 (3.00)	0.021	0.09-0.91

Additional items were analyzed from the survey regarding the relevance of this simulation to the participants' clinical practice. All participants (100% agree/strongly agree) felt the content of the simulation was relevant to their work, and 78% of participants felt the simulation was realistic, with the most common complaint of lack of understanding of which actions could be performed with the model. Facilitators were identified as knowledgeable (96.9% agree/strongly agree), responsive to participant needs (96.9%), and effective in helping participants learn new information (93.8%). The use of in-situ learning in the ER trauma bays was well received for this scenario as 96.9% (31/32) of respondents agreed or strongly agreed that the learning environment was conducive to learning. In addition, greater than 90% (29/32 agree/strongly agree) intended to use what they learned from the simulation in future practice (Table 4).

**Table 4:** Post-Simulation Return on Learning Questionnaire Likert Scale from Strongly Disagree to Strongly Agree. Percentages based on the number of participants who responded agree or strongly agree.

Post-Education Return on Learning Questionnaire	% Agree/Strongly Agree
This simulation provided me with new information (or clarified existing information)	96.88% (31/32)
I intend to use what I learned from this simulation	90.63% (29/32)
The simulation was realistic	78.13% (25/32)
The facilitator(s) was knowledgeable about the subject	96.88% (31/32)
The facilitator(s) was effective in helping me learn new information (or clarify existing information)	93.75% (30/32)
The facilitator(s) was responsive to participants' needs and questions	96.88% (31/32)
The learning environment was conducive to learning	96.88% (31/32)

Additionally, short answer questions were asked regarding strengths, weaknesses, and potential changes to the simulation. Selected responses are displayed in Table 5.

**Table 5:** Selected Learner Responses from Qualitative Feedback

Open Text Survey Questions	Selected Answers
Which part of this simulation did you find to be the most helpful? Why?	“The post-simulation discussion.” “The simulation itself was valuable but the debrief afterward discussing successes and opportunities for improvement was most helpful in ensuring everyone is on the same page.” “Multiple disciplines present during the same scenario to practice communication and shared responsibilities."
Are there any aspects of this simulation that you would change? If so, how would you improve it?	“Standardize available supply items in the trauma room.” “Explain the roles of each team at the beginning of debrief so other teams are comfortable and aware of what the other team is expected to do.”
Please provide us with suggestions for improving the content, facilitation, and delivery of this simulation.	Having sims more regularly. Also having live tours and reviews of different areas of the hospital as well as intermittent eval of equipment kits available esp those of interdisciplinary nature. More frequent sims to improve workflow and collaboration between specialties.

## Discussion

This study was performed for multi-disciplinary education due to a lack of simulation research regarding REBOA usage in the setting of pregnant trauma, despite the fact that many studies on pregnant trauma and REBOA have been published independently [2,3,5-9,26]. In addition, while there are good clinical indications for REBOA, there is data to suggest that there is an overall lack of reliable clinician comfort with doing so [27]. A significant reason for this is that the frequency of REBOA usage in actual patient care remains low [28]. One proposed solution to this has been the implementation of a formal REBOA training course, recommended to be taken every six months due to the reported decrease in subjective comfort level, to improve knowledge retention in the absence of clinical cases [27].

The only learning objective that showed a statistically significant improvement for physicians was identifying indications and contraindications of REBOA in a hemorrhaging patient. This objective was not significantly improved when applied to the learners as a whole. Of the physicians participating the emergency physicians, trauma attendings, and surgery residents are all ATLS certified; while the OBGYN residents and attendings are not. In contrast, three other objectives had significant improvement for the group as a whole but lacked significance when applied to physician learners only. Based on this result, it appears that the objective focused on REBOA indications and placement was an especially meaningful learning objective for the physicians participating in the training and was likely not in the scope of practice for other learners. This is likely because indications for and placement of REBOA are managed solely by physicians at the participants’ institution. The lack of significance in the objectives focusing on MTP, prioritization of care for pregnancy-related hemoperitoneum with shock, and decision-making in pregnancy-related trauma for the physician group likely reflects a higher initial comfort level with those objectives. Perhaps in part due to the frequent education residents receive regarding these topics [18,20]. While not statistically significant, all these objectives did show improved confidence for physician learners. In addition, with a smaller group of respondents, significance is more difficult to achieve due to limited power. The objective related to a comfort level with REBOA remained the least confident objective in both groups for the pre- and post-simulation survey, demonstrating its rare use and overall low level of comfort. Additional training will continue to be implemented to improve physician comfort with REBOA.

In the qualitative analysis of the case, participants highlighted the debriefing session at the conclusion of the simulation to help clarify the points of the case. The debriefing session was facilitated by leaders who had undergone formal debriefing training. Qualitative data was obtained from short answer questions at the conclusion of the questionnaire as well as written accounts of the debriefing discussion. One participant stated, “The simulation itself was valuable but the debrief afterward discussing successes and opportunities for improvement was most helpful in ensuring everyone is on the same page.” Other participants highlighted that “talking about communication between services" was most helpful. As noted above, this was an in-situ simulation conducted in the emergency department trauma bay. This allowed participants to discuss the layout of the space, equipment available, and established processes that were observed not only in this simulated case, but also through the debriefing, applying these to actual patient care. One observation that was noted and discussed during the debrief session was the instrument supply and organization of the emergency thoracotomy tray. This spawned a conversation about a future quality improvement project on restocking the tray with different equipment to facilitate more efficient patient care. Critiques of the simulation included improved audio volume for learners who were watching, more detailed explanations in the pre-brief setting on what tasks were able to be performed on the manikin, and running the simulation more frequently so that additional participants could be actively involved. Several participants expressed a desire for continued future simulations similar to that described herein involving a multi-disciplinary team caring for a critically ill or injured patient.

Given the high acuity of patients that require REBOA, comfort with identifying appropriate patients and proper placement of catheters in a timely manner is critical. Simulation-based learning allows providers to engage and collaborate with colleagues on how to approach this low-frequency high-risk scenario in a controlled environment. This interdisciplinary simulation-based education focused on creating a dynamic scenario that provided realistic patient outcomes based on the team’s decision-making. The case was designed to encourage participant interpretation of the patient’s clinical presentation, course of action for resuscitation, options for hemorrhage control, and ultimate disposition of the patient(s). As demonstrated by this study, utilizing a multidisciplinary in-situ simulation scenario resulted in a significant increase in providers’, and importantly physicians’, confidence level in the ability to recognize indications for using the REBOA catheter. All participants (100%) felt the study was relevant to their work, and a large majority of them felt the study was realistic and provided them with new information.

There are multiple limitations to this study. First, the sample size is small as the simulation was performed at a single institution and performed on a single day. Specifically, the physicians’ learner group is small relative to the total learners. Third, the Return on Learning questionnaire was only administered at the conclusion of the simulation activity, which may have introduced recall bias amongst participants when rating their confidence levels prior to the simulation. There were also no objective measures of competency related to the objectives, such as time for REBOA placement or correct and timely utilization of MTP. Due to the low-frequency nature of the scenario presented, follow-up application will be difficult to obtain and no participant identifiers were utilized during the initial survey to determine eligibility for follow-up data. No long-term retention of knowledge data was collected for this study. It may be difficult to replicate the exact simulator model used due to the expert staffing required for the creation of the model.

Future research should focus on expanding the role of simulation in obstetric trauma and REBOA usage with the aim of increasing knowledge on the utility of REBOA in this setting. Additionally, patient-centered outcomes such as morbidity and mortality should be evaluated as a result of this simulation-based training and changes that may result in increased frequency of REBOA utilization in this patient population. Additionally, a specific manikin manufactured to realistically recreate this simulation could also be explored.

This article was previously presented as an oral abstract at the Columbus Surgical Society Research Sharing Day, on May 10, 2022.

## Conclusions

A multidisciplinary in-situ simulation for the management of a pregnant patient in hemorrhagic shock secondary to penetrating abdominal trauma improved learner confidence in MTP, care of a pregnant patient in hemorrhagic shock, and abdominal trauma in pregnancy. Physician learners also gained confidence in the indications for REBOA placement in abdominal trauma. All learners felt this education was relevant to their work and most felt it was realistic and provided new information. A multidisciplinary in-situ simulation successfully provides education on low-frequency high-risk situations such as traumatically injured pregnant patients.

## Figures and Tables

**Figure 2:**
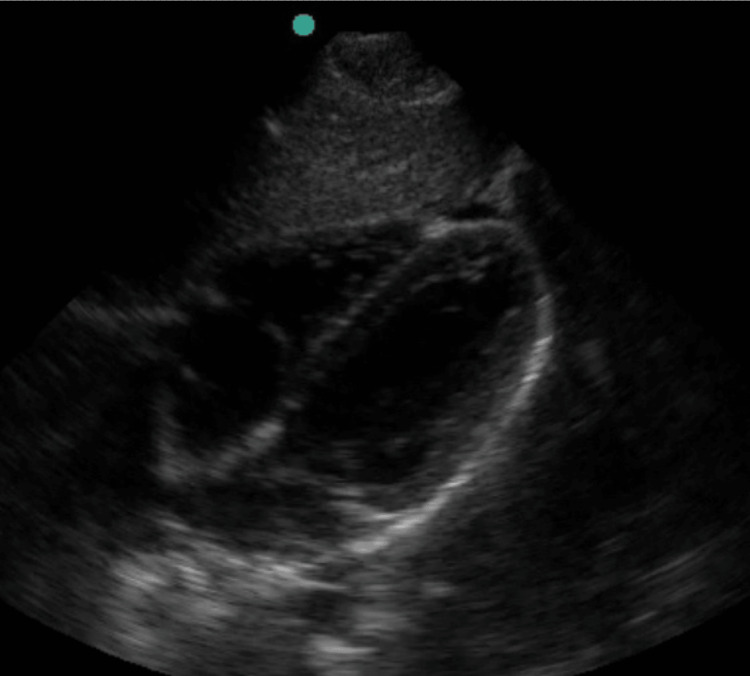
Subxyphoid Ultrasound Image

**Figure 3:**
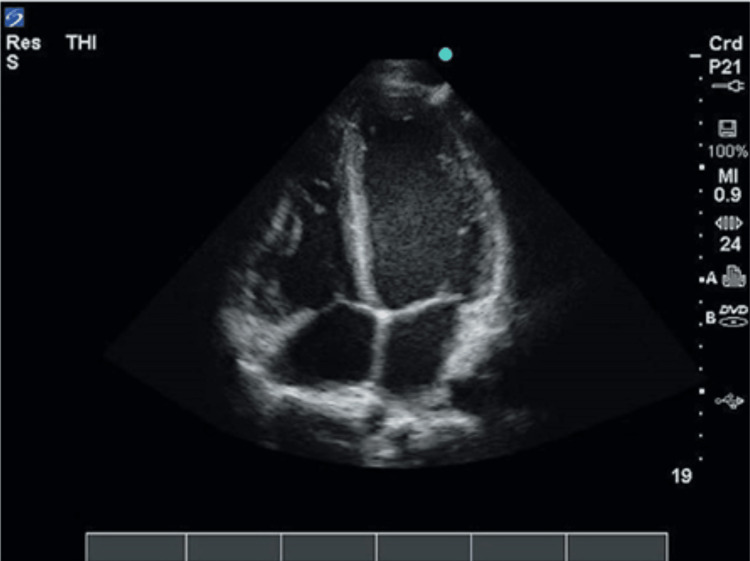
Apical Ultrasound Image FAST Exam FAST: Focused Assessment with Sonography in Trauma

**Figure 4:**
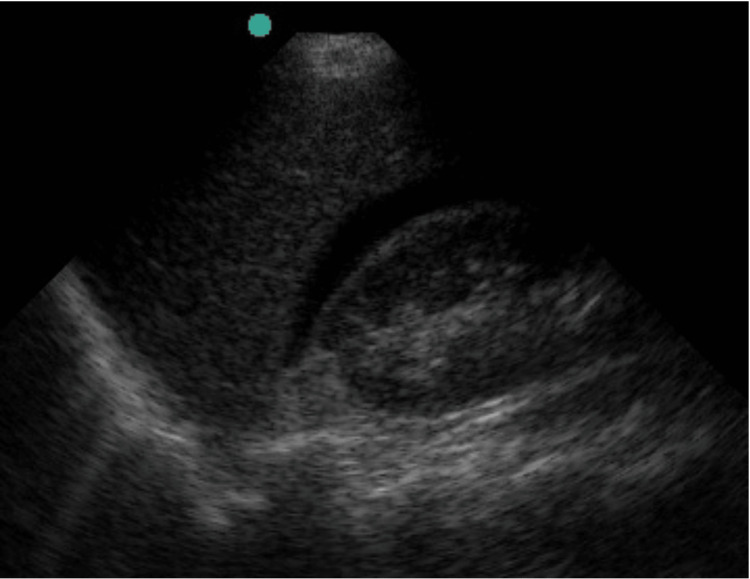
Right Upper Quadrant Ultrasound Image FAST Exam FAST: Focused Assessment with Sonography in Trauma

**Figure 5:**
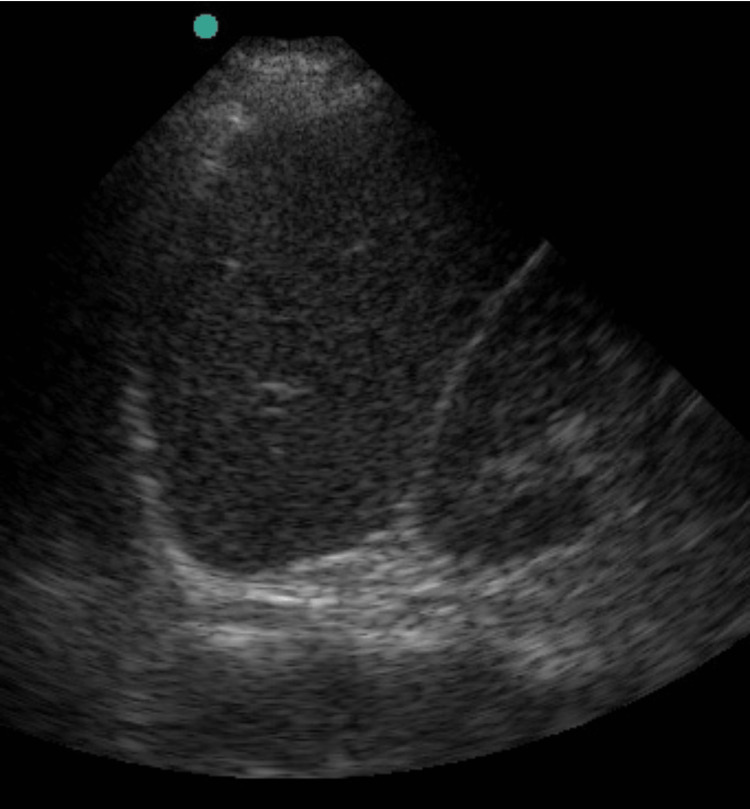
Left Upper Quadrant Ultrasound Image FAST Exam FAST: Focused Assessment with Sonography in Trauma

**Figure 6:**
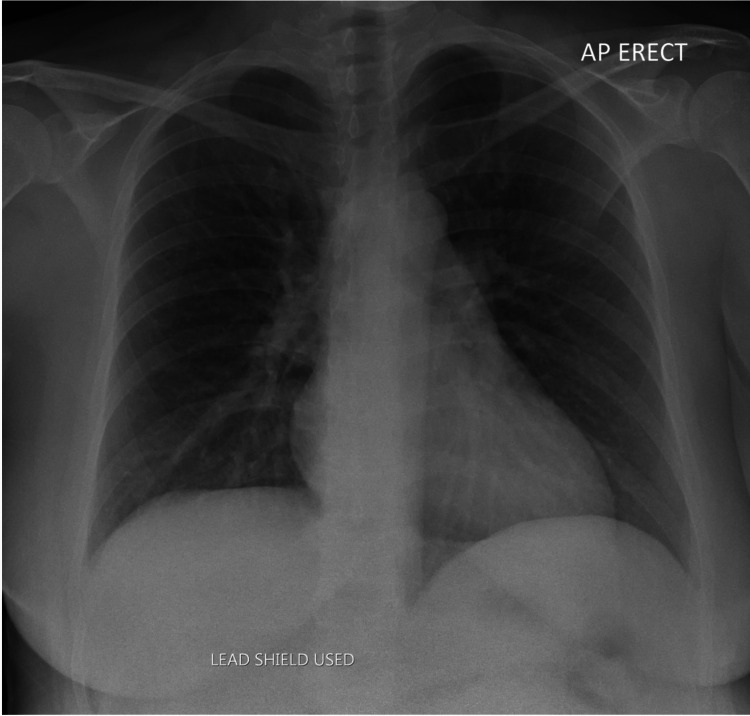
Chest X-ray

**Figure 7:**
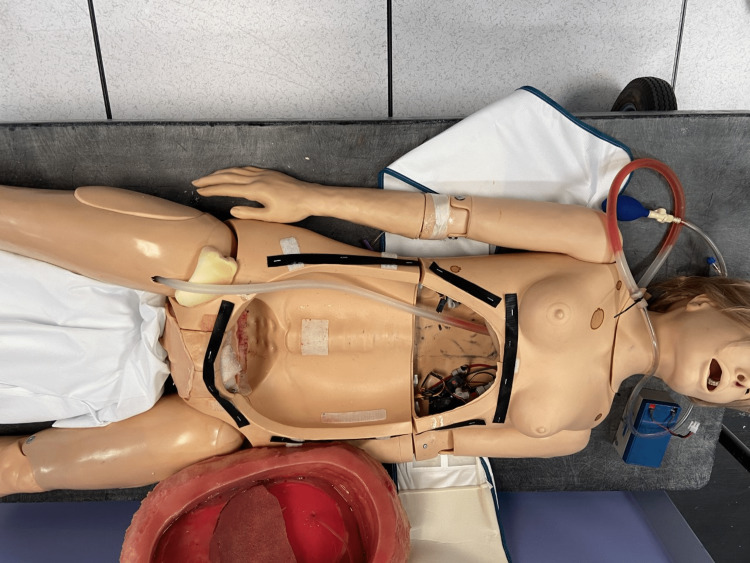
Noelle Manikin with Abdominal Aortic Tubing for REBOA Insertion REBOA: Resuscitative Endovascular Balloon Occlusion of the Aorta

**Figure 8:**
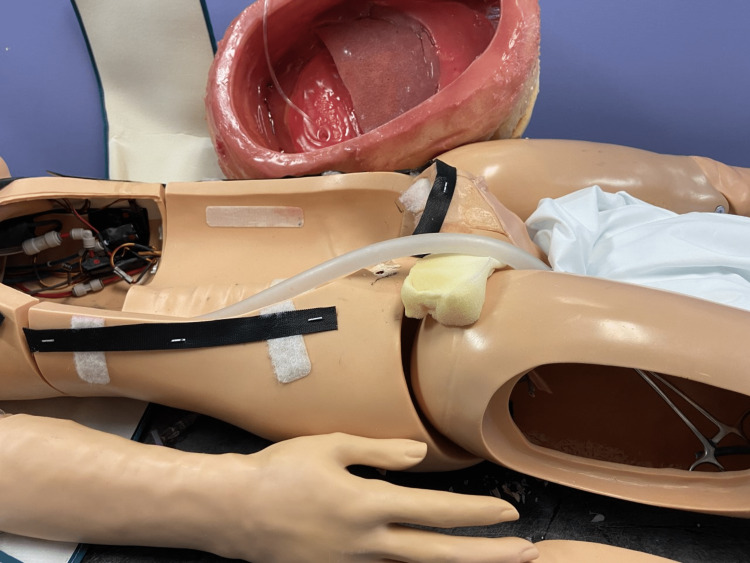
Side-view of Manikin Showing Femoral Access for REBOA Insertion REBOA: Resuscitative Endovascular Balloon Occlusion of the Aorta

**Figure 9:**
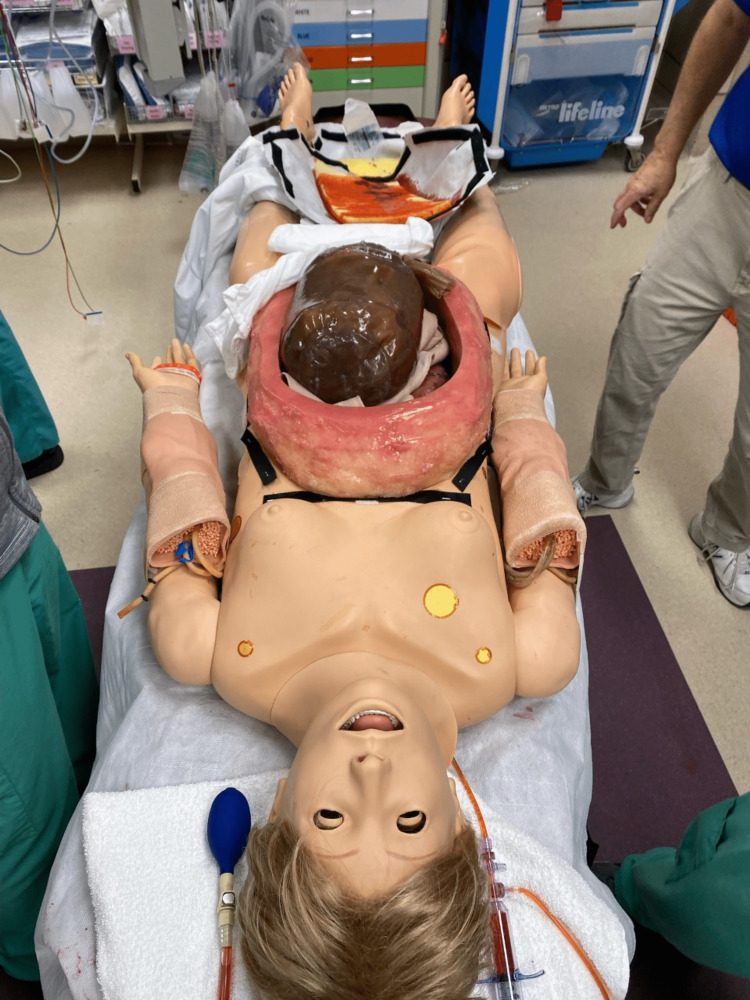
Manikin Pre-simulation with Filled Uterus and Abdominal Contents

**Figure 10:**
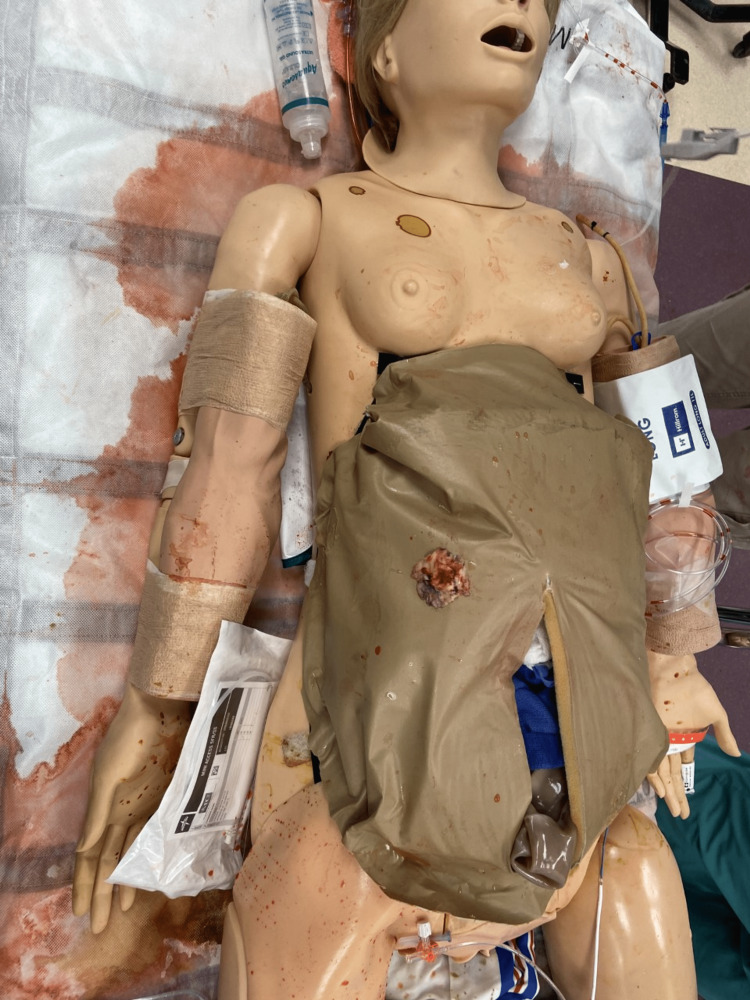
Manikin Post-simulation with Emergency Cesarean Section Performed

**Figure 11:**
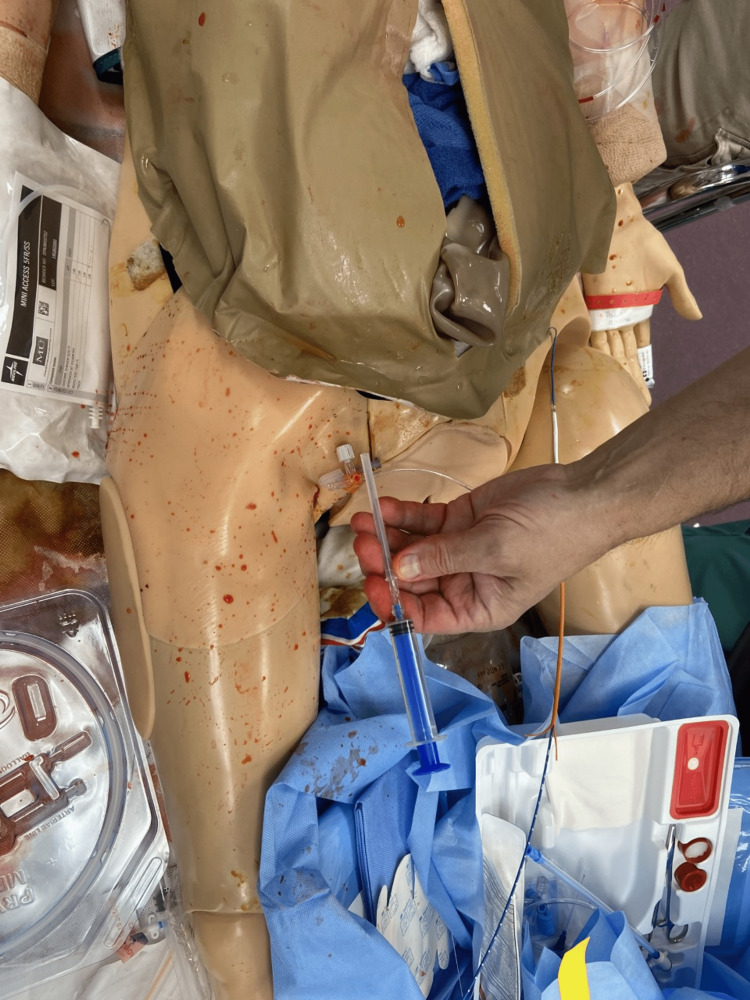
Post-simulation Manikin Demonstrating Femoral Access for REBOA Catheter REBOA: Resuscitative Endovascular Balloon Occlusion of the Aorta

## Appendices

OhioHealth learning scenario development form

Scenario Title: Abdominal GSW in Pregnant Patient

Target Learners: Physicians X Nurses X EMS X Other

Estimated Simulation Time: 15:00 Estimated Debriefing Time: 45:00 Estimated Total Time: 60:00

Curricular information

Primary Learning Objectives

By the end of this simulation experience learners will be able to:

1. Prioritize the care of a pregnant patient with hemorrhagic shock from hemoperitoneum

2. Demonstrate interdisciplinary collaboration with other specialties

3. Apply massive transfusion protocol (MTP) to the appropriate clinical settings

4. Analyze critical decisions made for abdominal trauma in pregnant female patients

5. Identify indications/contraindications for REBOA placement in hemorrhaging patient

Critical actions checklist

Complete Critical Action

☐ 1. Assess the mother and fetus and determine the need for emergency surgery

☐ 2. Appropriate operative approach for cesarean delivery

☐ 3. Recognize hemoperitoneum in the pregnant patient

☐ 4. Resuscitation of the patient in hemorrhagic shock (massive transfusion)

☐ 5. Evaluation of penetrating trauma as a source of bleeding

☐ 6. Appropriate management and disposition of patients with abdominal trauma shock

☐ 7. Appropriate deployment of REBOA for cessation of bleeding during operative delivery

Case narrative

Name: Gabby Sue Williams DOB: June 4, 1993 Age: 28 Sex: Female Weight: 195 Height: 5’ 7”

Chief Complaint: GSW to abdomen

History of Present Illness/Injury

Pt is a 28 yo G2P1 at 36 weeks who presents to ED by EMS following an argument with SO that resulted in GSW to the right upper abdomen. Now complaining of RUQ pain and blood loss. Bruising of arms but did not fall or hit head/stomach. Was initially normotensive at 135/80, but now hypotensive at 80/45 and HR in 120s. The emergency department called OB and General Surgery due to trauma in pregnancy. The patient's medical history is shown in Table 6.

**Table 6:** Patient's Medical History IPV - intimate partner violence

Medical History	
Past Medical: History of IPV Anxiety	Surgical History: Laprascopic cholecystectomy 2016
Medications: Prenatal vitamin Sertraline 100mg daily	Obstetrical History: 2015: Spontaneous Vaginal Delivery, uncomplicated 41 weeks
Allergies: No Known Drug Allergies	
Social History: Alcohol: Occasional when not pregnant Tobacco: None Illicit: +Marijuana	Family History: Mother: Breast cancer at age 65, Hypertension, hypothyroid Father: Hypertension, Hyperlipidemia, Diabetes Mellitus

Physical exam

GCS: Eyes: 2 Verbal: 4 Motor: 5 Total: 11

General: Unwell-appearing gravid female, pale

HEENT: NCAT

Cardiovascular: tachycardic; slight systolic ejection murmur

Pulmonary: CTA B; no wheezes/rhonchi/rales

Abdomen: GSW to the right upper abdomen, bleeding present, gravid 15cm above the umbilicus

Extremities: 1+ pulses

Skin: Cool and clammy; pale

Neuro: +2 dtrs throughout; no clonus

GU: Gravid uterus; c/w 36 weeks

Other: Fhts: 130s with moderate variability; occasional late decels; no accels

ROS: no recent illnesses, some lower extremity edema, otherwise at baseline health

Page Break

Discussion

Management of a pregnant patient who has sustained abdominal trauma requires a multidisciplinary approach. The handoff from ER to OB and Gen Surg is important. Examination of the fetus via heart tones is crucial. With the presence of decelerations, bleeding, and a mother who is becoming more hypotensive and less responsive, urgent cesarean delivery with surgical management of acute sharp impact trauma is important. Aggressive resuscitation of the mother with a massive transfusion protocol will be necessary and may require additional hemodynamic intervention such as REBOA placement. After the delivery of the fetus, the surgery team should assume care and attempt to stop the source of bleeding from a gunshot wound. Once hemostasis is obtained and surgical correction completed, the patient will ultimately need to be dispositioned to the OR for definitive management.

Consult(s)/clinical progression

OB/GS are called to ER to evaluate pregnant patient victim of abdominal GSW

The patient found to be in extremis with bleeding and FHT with decelerations

Surgery evaluates the abdominal cavity with a FAST scan for the source of bleeding secondary to GSW

OB determines the need for emergent cesarean section in ER

GS coordinates resuscitation and need for REBOA placement

The patient appropriately managed and dispositioned

Page Break

Instructor Notes

VOCERA trauma page script

Paged to L&D mom/baby, Trauma team

“This is a trauma drill. Incoming to trauma bay 1. 28-year-old female who is 36 weeks pregnant. Brought in by EMS after GSW to the right upper abdomen. Arrival time 1 minute. Repeat, this is a drill.”

Scenario begins with EMS having an empty bed AFTER transfer to ER cot and giving sign out to the incoming trauma and OB team. Table 7 outlines the clinical scenario branch points for the learners to work through. Table 8 demonstrates the environmental setup for the in-situ simulation.

**Table 7:** Case Scenario Branch Point Diagram Abbreviation Key: HR - heart rate, BP - blood pressure, O2Sat - Oxygen saturation percentage, Temp - temperature, RR - Respiratory rate, FHT - fetal heart tones, L&D - labor and delivery, GSW - gunshot wound, IVF - intravenous fluids, FAST - Focused Assessment with Sonography in Trauma, ER - emergency room, GCS - Glasgow Coma Scale, ACLS - advanced cardiac life support, ROSC - return of spontaneous circulation, OR - operating room, MTP - massive transfusion protocol, C/S - cesarean section, GS - general surgery, OB - obstetrics and gynecology, REBOA - Resuscitative Endovascular Balloon Occlusion of the Aorta

Scenario Branch Points, Modifiers, and Triggers			
Patient State	Patient Status	Learner Actions, Modifiers & Triggers to Next State	
1. Initial (in ER) Rhythm: HR: 119 BP: 80/45 RR: 18 O2Sat: 96 CO2: Temp: 97.8 Eyes: Lung Sounds: R: L: Heart Sounds: Bowel Sounds: GSW to right upper abdomen	Patient moaning, opens eyes to pain	Learner Actions: Learners will make an initial assessment of the patient. Assess FHT and need for urgent operative management	Modifiers: If learners don’t request tocometry, the attending or L&D nurses will prompt
			Triggers: After 3 minutes, if no plan has been made, patient will fully lose consciousness, trigger pulselessness and will need resuscitation and emergent intubation (step #2). If learners request IVF and plan to urgently intubate with FAST scan go to branch #3

Scenario Branch Points, Modifiers, and Triggers			
Patient State	Patient Status	Learner Actions, Modifiers & Triggers to Next State	
2. ER post loss of consciousness GCS 8 Rhythm: NO pulse HR: 130 BP: 70/40 RR: 20 O2Sat: 95 CO2: Temp: Eyes: Closed Lung Sounds: R: L: Heart Sounds: Bowel Sounds:	Patient acutely decompensated secondary to blood loss and will need to be intubated.	Learner Actions: Emergently intubate and begin ACLS protocol. ROSC after 1 cycle	Modifiers: Attending suggests patient not stable enough to transfer to OR if that is suggested
			Triggers: Learners will be prompted to plan for emergent ER C-section and hemostasis actions

Scenario Branch Points, Modifiers, and Triggers			
Patient State	Patient Status	Learner Actions, Modifiers & Triggers to Next State	
2. ER s/p IVF identified need for intubation Rhythm: HR: 130 BP: 70/40 RR: 20 O2Sat: 95 CO2: Temp: Eyes: Closed Lung Sounds: R: L: Heart Sounds: Bowel Sounds:	Patient stable but critical secondary to blood loss and will need to be intubated.	Learner Actions: Learners should evaluate intubate and assess fetal heart tones, seeing minimal variability and late decelerations. Plan for emergent surgery	Modifiers: Attending suggests patient not stable enough to transfer to OR if that is suggested
			Triggers: Learners will be prompted to plan for emergent ER C-section. Proceed to step 4.

Scenario Branch Points, Modifiers, and Triggers			
Patient State	Patient Status	Learner Actions, Modifiers & Triggers to Next State	
4. Intubated in the ER Rhythm: HR: 108 BP: 96/55 RR: 15 O2Sat: 96 CO2: Temp: Eyes: Lung Sounds: R: L: Heart Sounds: Bowel Sounds:	Patient intubated and sedated	Learner Actions: Learners (GS) will perform FAST scan to determine site of bleeding and appropriate action Start MTP OB learners will then determine need for emergent C/S in ER	Modifiers: Attending will prompt to do vertical incision
			Triggers: Will proceed to incision with plan for REBOA for hemostasis. If no MTP or other blood product or hemostatic action taken, patient to become pulseless and require resuscitation (vitals #6). Otherwise, proceed to step 5

Scenario Branch Points, Modifiers, and Triggers			
Patient State	Patient Status	Learner Actions, Modifiers & Triggers to Next State	
5. ER – after incision Rhythm: HR: 118 BP: 88/47 RR: anesthesia O2Sat: 98 CO2: Temp: 97.8 Eyes: Lung Sounds: R: L: Heart Sounds: Bowel Sounds:	Patient under general anesthesia	Learner Actions: Upon entering the abdomen learners will encounter additional blood. OB will proceed to c-section and deliver fetus. GS will consider concurrent vs pre- vs post-C/S REBOA placement	Modifiers: If learners evaluate uterus without commenting on hemoperitoneum, attending will prompt. Surgery attending will discuss sources of bleeding as residents evaluate (clarify anything atypical due to simulation or may comment that pressure is dropping
			Triggers: If no plan for hemodynamic stability (i.e. wound packing, REBOA placement, direct pressure) patient to become overtly hypotensive and go pulseless during C/S (vitals #6). Otherwise proceed to step 7

Scenario Branch Points, Modifiers, and Triggers			
Patient State	Patient Status	Learner Actions, Modifiers & Triggers to Next State	
6. ER – uncontrolled bleeding Rhythm: pulseless HR: 150 BP: Undetectable RR: X O2Sat: 97 CO2: Temp: Eyes: Lung Sounds: R: L: Heart Sounds: Bowel Sounds:	Under general anesthesia	Learner Actions: ACLS protocol performed by GS while OB attempts to deliver fetus in timely manner	Modifiers
			Triggers: ROSC with one cycle of compressions and defibrillation to step #4

Scenario Branch Points, Modifiers, and Triggers			
Patient State	Patient Status	Learner Actions, Modifiers & Triggers to Next State	
7. ER – bleeding eval Rhythm: HR: 148 BP: 68/36 RR: anesthesia O2Sat: 96 CO2: Temp: 97.0 Eyes: Lung Sounds: R: L: Heart Sounds: Bowel Sounds:	General anesthesia	Learner Actions: Learners will need to coordinate resuscitation. Continue MTP and rapid infusion. Learners will identify liver as source of bleeding and ensure REBOA placement with inflation of balloon in Zone 1. Learners will evaluate for other sources of bleeding (spleen, retroperitoneum)	Modifiers: Will be prompted by anesthesia and/or surgery attending that patient is decompensating
			Triggers: If learners identify bleeding and inflate REBOA or pack area proceed to vitals step #8

Scenario Branch Points, Modifiers, and Triggers			
Patient State	Patient Status	Learner Actions, Modifiers & Triggers to Next State	
8. ER – definitive management Rhythm: HR: 118 BP: 88/46 RR: anesthesia O2Sat: 97 CO2: Temp: Eyes: Lung Sounds: R: L: Heart Sounds: Bowel Sounds:	Under general anesthesia	Learner Actions: Massive transfusion will be started. REBOA will be placed and fetus delivered. Learners will discuss moving to OR for definitive surgical management of GSW and closure of C/S	Modifiers
			Triggers Completed

**Table 8:** Scenario Environment Planning Sheet FAST- Focused Assessment with Sonography in Trauma CXR - Chest X-ray REBOA - Resuscitative Endovascular Balloon Occlusion of the Aorta GS - General Surgery OB - obstetrics and gynecology ER/EMS - Emergency room and Emergency Medical Services OR - operating room A/V - audio visual

Environment
Patient Props: Manikin
Room Props: Ultrasound machine - FAST images CXR image available Beats Pill Vitals screen REBOA Baby cart Tocometer with display Homemade REBOA model Medications: Fentanyl
Simulation Room Type: Emergency Department Trauma Bay 1
Distractors:
Staffing/Confederates: ER/EMS for handoff, OR nurses, GS/OB residents, simulation staff for mannequin and A/V assistance, debriefers

Focused Assessment with Sonography in Trauma (FAST) images** **(Figures 2, 3, 4, and 5).

Chest X-ray was provided for the simulation as well (Figure 6).

Baby Report

LPI baby to a 28 yo G2P1 now 2 mother after 36+1 weeks via C-section without labor due to maternal hemorrhage s/p GSW. Maternal blood type O+, Ab negative. GBS negative, Hepatitis B negative, RPR/Syphilis Ab Non-reactive or negative, HIV negative, Rubella Nonimmune. APGARS 6/?? . AROM, at delivery, clear

EMS handoff

Name: Gabby Sue Williams DOB: June 4, 1993 Age: 28 Sex: Female Weight: 195 Height: 5’ 7”

Chief Complaint: GSW to abdomen

History of Present Illness/Injury

Pt is a 28 yo F at 36 weeks who presents following an argument with significant other that resulted in GSW to the right upper abdomen. Now complaining of RUQ pain and blood loss. Bruising of arms but did not fall or hit head/stomach. Was initially normotensive at 135/80 when EMS arrived, but she became hypotensive to 80/45 with HR in 120s. Initial fetal heart tones were in the 170s.

PIV placed and IV NS running.

Photos of simulation model.

Figures 7 and 8 demonstrate the base Noelle manikin with the improvised aortic and iliac artery that was used for REBOA insertion.

Figure 9 demonstrates the model with intestines, 3D printed liver, and uterus filled with water and doll prior to the simulation with the abdominal skin pad removed.

Figures 10 and 11 show the manikin post-simulation after emergency C-section and REBOA placement, with additional highlighting of the REBOA insertion in Figure 11.
